# Barriers and facilitators to weight-loss in patients with overweight/obesity and cardiac disease: a realist qualitative synthesis

**DOI:** 10.1080/17482631.2024.2419574

**Published:** 2024-10-26

**Authors:** Rachel Bates, Cara Bailey, Anne Topping

**Affiliations:** a Oxford Heart Centre, Oxford University Hospitals NHS Foundation Trust, Oxford, UK; b Institute of Clinical Sciences, University of Birmingham, Edgbaston, Birmingham, UK; c School of Nursing, AHPs and Midwifery, University Hospital Birmingham NHS Foundation Trust, Birmingham, UK

**Keywords:** Cardiovascular disease, myocardial infarction, overweight, obesity, cardiac rehabilitation, weight loss, COM-B, realist thematic synthesis

## Abstract

**Purpose:**

Lifestyle advice for cardiac patients with overweight/obesity includes weight management, yet few achieve significant weight loss. People with heart disease may require different support to the general population. We synthesized evidence that reported cardiac patients’ weight management experiences to identify barriers and facilitators to weight loss.

**Methods:**

Our realist review identified five manuscripts reporting four studies of weight management experiences of people with heart disease. The capability, opportunity, motivation behaviour change model (COM-B) provided the framework for thematic synthesis.

**Results:**

The studies included qualitative data from 117 participants and revealed factors favouring or impeding effective weight management during cardiac rehabilitation (CR) and in participants’ daily lives. We identified four major themes illustrating participants’ wish to change harmful health behaviours, adopting exercise and psychological strategies to facilitate change, social and professional support, and regaining control after a cardiac event. These themes broadly aligned with the COM-B categories of capability, opportunity, and motivation.

**Conclusions:**

Most cardiac patients with overweight/obesity express a desire to lose weight. Participants preferred personalized services that could adapt to meet their individual needs, but struggled to make sustainable changes for interconnecting psychological, social, cultural, and financial reasons. Consideration of these complexities when designing programmes may help to support successful weight management.

## Introduction

Lifestyle advice for cardiac patients with overweight/obesity normally includes weight management due to the beneficial effects on cardiac risk factors and comorbid conditions (NHS; Powell-Wiley et al., 2021). Obesity leads to the development of cardiovascular disease independently of other cardiovascular risk factors. Overweight/obesity contributes directly to hypertension, dyslipidaemia, type 2 diabetes mellitus, and obstructive sleep apnoea, all of which are risk factors associated with cardiovascular disease (Powell-Wiley et al., 2021).

Evidence from studies of cardiac rehabilitation (CR) shows few participants achieve long-term weight-loss (Gomadam et al., 2016; Hushcha et al., 2022). Studies of CR and weight management for cardiac conditions frequently report mean weight loss but omit the range of change across participants (Abed et al., 2013; Hassan & Nahas, 2016; Hushcha et al., 2022; Rejeski et al., 2011; Zhang et al., 2018). Significant weight loss in a minority of participants for whom an intervention is particularly effective may thus disguise poorer outcomes experienced by a majority. Studies which categorize weight change show most participants’ weight remains unchanged or increases (Gomadam et al., 2016; Pathak et al., 2015; Tijssen et al., 2021). For example, in a study involving 1320 CR attendees, the reported weight loss achieved by 25% resulted in an overall decrease in mean weight of 3%, whereas 65% of patients achieved no weight loss and 10% weight gain (Gomadam et al., 2016). Further, a randomized controlled trial of a secondary prevention programme for patients with coronary artery disease reported weight gain in 36% of intervention participants and 41% of controls (Tijssen et al., 2021). Weight-loss attempts which result in fluctuating weight may adversely affect cardiometabolic risk factors (Pathak et al., 2015). Fluctuating weight in patients with atrial fibrillation (AF) participating in a weight management intervention was associated with increased antihypertensive medication requirements, higher fasting insulin and serum low-density lipoprotein levels, and adverse cardiac remodelling (Pathak et al., 2015). Better understanding of views and experiences of this majority is required to address barriers to weight management, avoid harm, and support better patient outcomes by informing the development of more effective programmes.

Barriers to weight-loss in the general population are well documented and include temptation, eating for pleasure, habitual behaviours, financial and time pressures, lack of professional and social support, stress, boredom, demotivation, among others (Fischer et al., 2020). Few qualitative studies explore the weight management experiences of people with heart disease, who may experience additional barriers related to their pathology. This review adopted a realist synthesis approach (Pawson, 2006) to explore weight-management experiences of cardiac patients with overweight/obesity with the aim of identifying clinically usable findings regarding barriers and facilitators to weight management in this population. The COM-B model of behaviour change (Michie et al., 2011, 2014) was chosen as an analytical framework as it is widely used in UK health policy design to identify which interventions will enable behaviour change.

## Methods

### Design and theoretical basis

The RAMESES guidelines for realist syntheses (Wong et al., 2012) were used to systematically identify, extract, evaluate, and synthesize data from included qualitative studies to answer the question “What are the views and experiences of people with overweight/obesity and heart disease regarding weight management?”. The search and synthesis followed a predetermined protocol (appendix 1).

A realist review design was selected as particularly useful for interrogating complex interventions such as weight management programmes. Realist inquiry investigates the relationships between context, mechanism, and outcomes of health interventions with the aim of drawing usable, data-driven conclusions regarding what works for whom, how, and in which circumstances (Pawson, 2006; Rycroft-Malone et al., 2012). Here, the aim is to investigate the multifarious factors that inhibit weight management despite well-founded evidence of health benefits.

**Figure 1.**
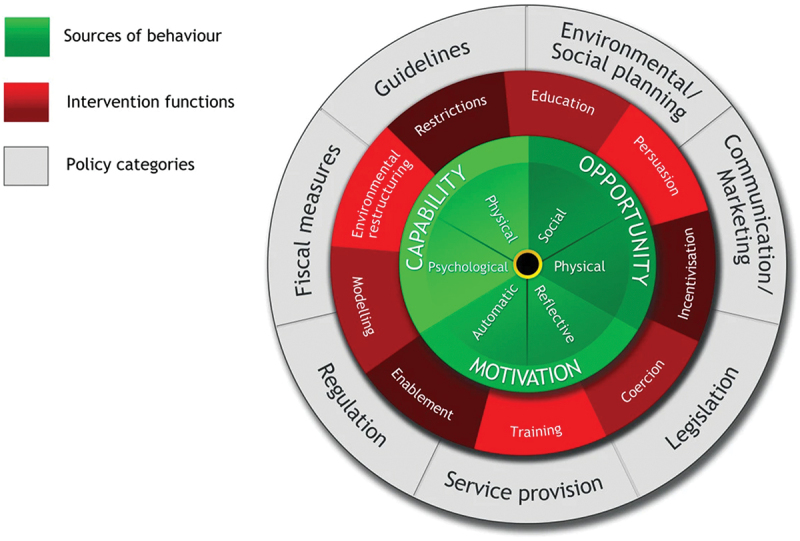
The behaviour change wheel (Michie et al., 2014).

As behaviour change is central to weight management, the COM-B model (Michie et al., 2011) (Figure 1) was used to guide the analysis. COM-B forms the hub of the “behaviour change wheel” (BCW), an intervention design method which involves identifying sources of health risk behaviour to target. According to the COM-B model, behaviour change (B) is dependent on the capability (C), opportunity (O), and motivation (M) of the target population to effect change. It has become the conceptual model underpinning various public health initiatives in England targeting obesity and health behaviour change (Public Health England, 2018, 2019, 2020). In this review, COM-B provided the framework to capture facilitators and limitations of weight-loss interventions by comparing programme aims with participants’ experiences.

### Search strategy

A key word search was conducted using the CINAHL, Medline, PubMed, PsycInfo, and GoogleScholar databases. Grey literature and conference proceedings were searched for non-peer reviewed studies which might contribute insights (further information on the search strategy can be found in Appendix 1). Search terms were developed using the PICo tool for qualitative research (Lockwood et al., 2015) (Table I).

**Table I.** PICo search terms.

Population	Interest	Context
People with heart disease and obesity	Views and experiences	Weight management
Search terms: Heart Heart disease Heart Condition Cardiac Cardiovascular Overweight Obese/Obesity/Obes* Arrhythmia High BMI	Search terms: Views Opinions Attitudes Perspectives Perceptions Barriers Obstacles Challenges Facilitators Experiences Want/desire Difficulty	Search terms: Weight management Weight loss Dieting Slimming Exercise Lose/losing weight Weight control Healthy weight Bariatric surgery Gastric band/balloon/bypass

A total of 1090 titles were identified from the five databases (of which 80 duplicates were removed) and 2 reports were identified from other sources. No existing reviews were identified. The studies were screened according to predetermined eligibility criteria. To enable online sharing of information during the analysis period which coincided with service disruption cause by the COVID-19 pandemic when virtual collaboration was still at a fledgling stage, sources were managed using Google Sheets.

### Eligibility criteria

- Original qualitative or mixed-methods designs, reporting participants’ views of weight management
- In order to increase the transferability of findings to the UK system, studies conducted in western societies comparable to the UK in Europe, Australasia, or North America were included
- Participants had acquired (as opposed to congenital) heart disease and were overweight or obese or had successfully lost weight, as defined by the study. Heart failure was excluded due to the confounding effects of diuretics for fluid management on weight measurement.
- Full-text available
- Studies not reporting participants’ experiences were excluded

**Figure 2.**
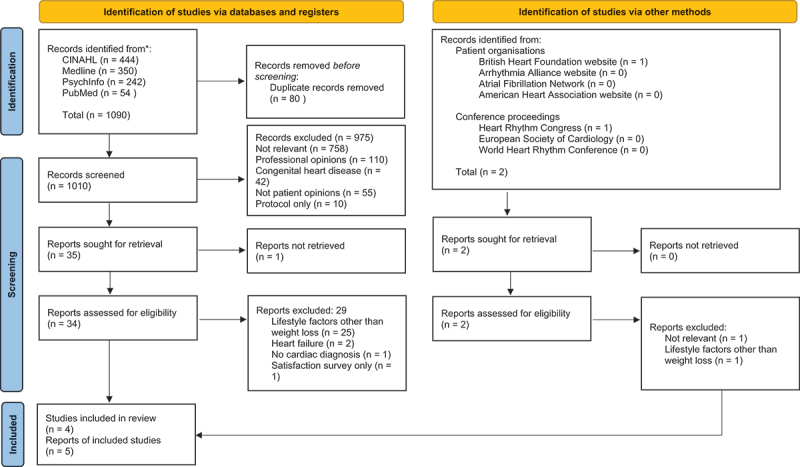
PRISMA flow chart (Page et al., 2021).

All papers were assessed by title (*n* = 1012) and abstract (*n* = 146) by RB and the process checked by CB and AT. Those not fulfilling the inclusion criteria were eliminated. Full-texts of the remaining papers (*n* = 34) were assessed by RB and AT. Any disagreements were resolved by CB. Five papers were included in the final analysis (Figure 2), representing a total of 117 participants across four countries (Australia, Canada, France, USA). Two papers (Gallagher et al., 2012a, 2012b) reported the same patient cohort. One paper (Génolini et al., 2013) was translated into English from the original French by RB and checked for accuracy by a French native-speaker with experience in the fields of sociology and qualitative research.

### Quality appraisal

The CASP checklist (2018) was used to evaluate the selected studies so the credibility of their findings could be appropriately balanced within the review. Three papers were considered to be high quality (Gallagher et al., 2012b; Kramer-Kile, 2013; Nadarajah, 2012), one medium (Gallagher et al., 2012a), and one limited due to poor reporting (Génolini et al., 2013) (Table II). No studies were excluded on the basis of methodological rigour as all offered valuable perspectives and rich, descriptive data (Carroll et al., 2012; Thorne, 2011).

**Table II.** Findings of critical appraisal skills programme (CASP) (2018) evaluation.

Study	Are the results of the study valid?						What are the results?			Will the results help locally?
	1	2	3	4	5	6	7	8	9	10
Gallagher et al. 2012a	Y	Y	Y	CT	Y	N	CT	CT	Y	Limited
Gallagher et al. 2012b	Y	Y	Y	Y	Y	N	CT	Y	Y	Yes
Génolini et al. 2013	CT	Y	CT	CT	N	N	N	N	CT	Very limited
Kramer-Kile, 2013	Y	Y	Y	Y	Y	Y	Y	Y	Y	Yes
Nadarajah, 2012	Y	Y	Y	Y	Y	N	Y	Y	Y	Yes

Key: Y=Yes, N=No, CT=Can’t Tell.

1. Clear aims 2. Appropriate methodology 3. Appropriate design 4. Appropriate recruitment strategy 5. Appropriate data collection 6. Consideration of researcher/participant relationship 7. Ethics considered 8. Rigorous data analysis 9. Clear statement of findings 10. Value of research.

### Data extraction and analysis methods

Initial data extraction identified weight management intervention types, numbers, demographics, and diagnoses of participants (Table III). Themes were identified by comparing the objectives of interventions with researchers’ interpretations and participant quotes. Coding and analysis were supported by NVivo 12 using framework method (Gale et al., 2013). The COM-B components capability, opportunity, and motivation were employed as the framework of pre-existing concepts that enabled coding. Realist review questions (what works for whom, how, to what extent, and under what circumstances?) facilitated a second-level interrogation to surface additional themes to contribute to the overall framework (Table IV). This systematic consideration of study findings from a theoretical behaviour change perspective (COM-B) and realist gaze provided flexibility to detect and characterize divergent themes and limitations (Dixon-Woods, 2011).

**Table III.** Characteristics of included studies.

Study and setting	Participants	Exclusions	Interventions and COM-B category	Outcomes	Study design	Theoretical basis
Gallagher et al. (2012a and 2012b) Sydney, Australia. Group based weight-loss programme adjunct to CR.	*N* = 35. BMI 27-38 with CVD or multiple CVD risk factors. Mean age 62. 71% male, 89% caucasian. Recruited from CR by invitation letter.	Unable to exercise, unable to speak English, cognitive impairment	Biweekly exercise sessions (C, O). Four information sessions in 16 weeks (C). Three telephone calls over next 8 months (M). Promotion of healthy eating, exercise and problem solving to achieve sustainable weight-loss (C, M). Pedometers (M).	Completed at least 16 weeks of programme. 66% of participants lost weight.	Focus group discussion on managing weight while participating in CR. Thematic analysis. Interviews conducted on completion of CR.	Inductive, interpretive.
Génolini et al. (2013) France. Cardiac risk-factor screening and CR.	*N* = 40 cardiac patients (57 obese patients and unspecified number of staff interviewed but not included in synthesis), BMI 30-40. Recruitment strategy not reported.	None listed.	No information on interventions reported.	Not reported	Observation and biographical interview, focus not reported. Thematic analysis. Timing of interviews not reported.	Sociology of socialization and social interaction
Kramer-Kile, (2013) Toronto, Canada. Two outpatient and one home-based CR programme.	*N* = 32. Diagnosis of CVD and DM2. Age 52-79. BMI range not reported. 52% male. Recruited from CR by poster.	Unable to speak English.	Structured exercise (C, O). Education on dietary management, weight-loss, smoking cessation, psychological support, and the management of co-morbidities (C)	Not reported	Semi-structured interview on diabetic management and weight loss. Thematic analysis. Two interviews conducted one month apart during or on completion of CR programme.	Sociology of corporeal realism and social theory of the body (Shilling, 2003)
Nadarajah, (2012) Maryland, USA. Outpatients CR programme	*N* = 10. Diagnosis of CVD. Age 46–75. BMI range not reported. Recruited from CR by invitation letter.	Psychometric test used to screen out patients with high stress and distress scores from the study.	Twelve week programme of exercise (C, O), education, risk factor assessment and reduction, pharmaceutical therapy optimization, counselling (C).	100% of participants lost weight.	Semi-structured interview on effect of cardiac event on physical, psychological, social and spiritual aspects of participants’ lives. Thematic analysis.	Biopsychosocial model (Engel, 1977) adapted by researcher to psycho-social-spiritual healing

Abbreviations: BMI Body Mass Index—CR Cardiac Rehabilitation—CVD Cardiovascular Disease—DM2 Diabetes Mellitus Type 2 – C Capability—O Opportunity—M Motivation.

**Table IV.** Examples of coding and framework analysis.

Quote (Study)	Initial code(s)	Notes	Re-coded to	COM-B category	Who/what/how/circumstances	Theme
Straightforward strategies, such as reducing plate size, were not only effective for portion control and time management, but also gave participants control over dietary intake, without family and friends necessarily being aware (Gallagher et al. 2012b)	Diet	Keeping advice simple and breaking down into do-able steps. Minimizing inconvenience or discomfort of others.	Simplifying complexity; Social meaning of food; Disrupting relationships	Capability; Opportunity	How—making the task manageable. Circumstances—accommodating changes within the family	Building capacity; Having control; Having support
“Going back to Grandma times, when she used to do the cooking with the most basic of the stuff.” (Gallagher et al. 2012b, p22)	Diet	Identifying previous, healthier eating habits with positive family associations	Maintaining identity	Motivation	Who—able to align personal values with those of CR	Wanting to change
He did not consider his first MI to be a serious event, but rather a mild warning sign that he needed to take his health more seriously. (Kramer-Kile, 2013, p103)	Understanding gravity	Several accounts refer to a heart attack not being serious, because treatment is quick and minimally invasive. This affects patients’ estimation of gravity and subsequent motivation to change behaviour.	Conceptualizing health and illness	Motivation	Who—able to accept diagnosis	Wanting to change
Rehabilitation immerses the patient and insulates them from the pressures of everyday life (stress, unhealthy lifestyle). *La rééducation (C2) immerge le patient et l’isole des contraintes de la vie quotidienne (stress, mauvaises habitudes d’hygiène, etc.)* (Génolini et al. 2013, p56).	Safe place	CR provides “safe” place to learn skills and form new habits. Residential CR takes further step by disrupting patient’s social context.	Re-learning; Disrupting relationships	Opportunity	What works—having opportunity to break with old habits	Having control
“And yes, it (cardiac rehabilitation) is expensive if you think about four times a week, you know. That can cost $300 dollars a month.” (Nadarajah, 2012, p74)	Cost	Many participants in private systems did not complete all stages of rehab because of cost. Insufficient time/support for new exercise/diet habits to become ingrained.	Socio-economic factors	Opportunity	What works—access to affordable services	Having support
The formation of autonomy differs greatly between institutions. During diagnosis, the cardiovascular risk “negotiations” are sensitive to the cultural similarity between the doctor and the patient. La formation à l’autonomie est très différente selon les institutions. Dans le diagnostic, les « négociations » autour du risque cardiovasculaire sont sensibles à la proximité culturelle entre le médecin et le patient.	Becoming independent	To maintain changes, patients need to develop independence. Degree to which they relate to medical staff affects acceptance of care/advice.	Developing independence; Maintaining identity; Socio-economic factors	Capability; Motivation; Opportunity	Who—able to align personal values with those of CR. How—assimilation of medical model by patient	Wanting to change; Having control

## Results

The five papers included 117 patients with cardiovascular disease (CVD) who had suffered acute coronary syndrome (ACS—myocardial infarction or unstable angina) and subsequently participated in a cardiac rehabilitation (CR) programme (see Table III for selected demographics). No eligible studies were identified that investigated weight management in patients with other cardiac pathologies, nor any that explored weight management interventions outside formal CR. All participants in Kramer-Kile (Kramer-Kile, 2013) had type 2 diabetes mellitus. Génolini et al (Génolini et al., 2013) (*n* = 40) did not report demographic characteristics. The remaining studies reported age range 46–79 years and included 30 female and 47 male participants. Ethnicity was reported by Nadarajah (Nadarajah, 2012) and Gallagher et al (Gallagher et al., 2012a, 2012b), respectively, as 90% and 89% Caucasian; Kramer Kile (Kramer-Kile, 2013) reported 81% of female and 56% of male participants identified as immigrants. BMI ranges were reported by Gallagher etl al (Gallagher et al., 2012a, 2012b) (27–38) and Génolini et al (Génolini et al., 2013) (30–40). Kramer-Kile (Kramer-Kile, 2013) and Nadarajah (Nadarajah, 2012) did not report BMI range.

### Principles of cardiac rehabilitation (CR)

The five studies described seven CR programmes. All aimed to reduce participants’ cardiac risk-factors by affecting behaviour change through health education, increasing exercise capacity, and improving psychological resilience (Gallagher et al., 2012a, 2012b; Génolini et al., 2013; Kramer-Kile, 2013; Nadarajah, 2012). Only two studies (Gallagher et al., 2012a, 2012b) identified weight-loss as a specific objective of CR, whereas participants in all studies were reported to cite weight loss as their desired outcome from CR (Gallagher et al., 2012a, 2012b; Génolini et al., 2013; Kramer-Kile, 2013; Nadarajah, 2012).

Where reported in full, CR interventions were categorized using COM-B (Table III). Most focussed on capability, aiming to educate participants in nutrition, build physical exercise capacity, and develop strategies to overcome challenges. Service provision and referral to CR represented the opportunity for participants to engage in behaviour change. Only one programme (Gallagher et al., 2012a, 2012b) included a specific motivational intervention (pedometers) but many participants reported gaining motivation from staff and peers.

### Thematic summary

**Figure 3.**
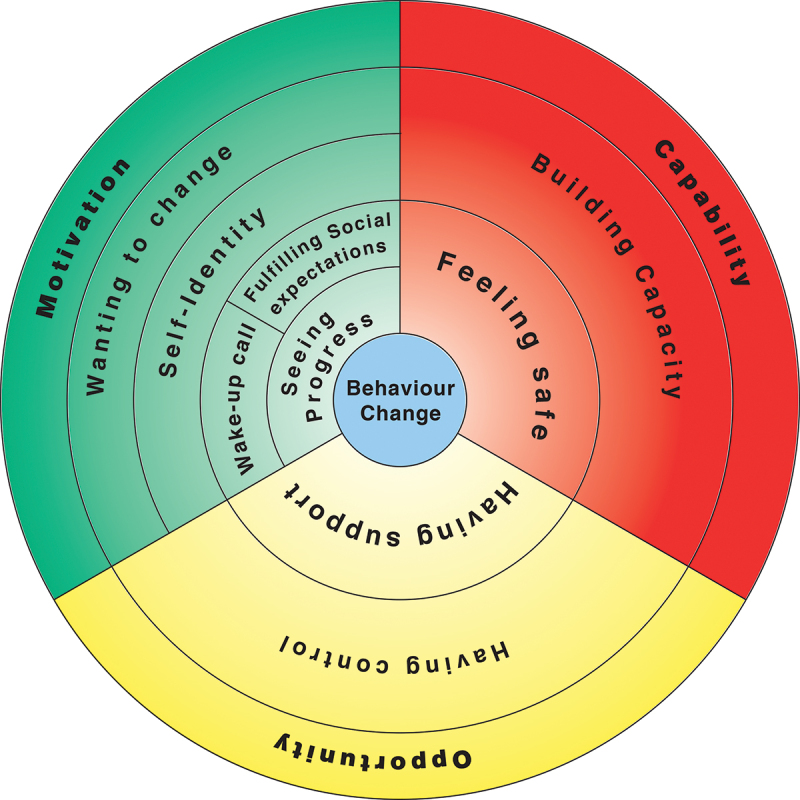
Diagram showing relation of themes to the COM-B model (Michie et al., 2011).

Four main themes were identified: *Wanting to change*; *Building capacity*; *Having support; Having control*. These themes broadly correspond to the COM-B categories of capability (*Building capacity*), opportunity (*Having control* and *Having support*) and motivation (*Wanting to change*). Various sub-themes emerged around the experience of ACS, the role of self-identity, social expectations, measuring progress, and the need to feel safe (Figure 3). These themes illustrate the interventions and conditions which most benefit participants, where interventions could be improved, and identify external factors that impact on the effectiveness of CR.

### Theme 1. Wanting to change

Unsurprisingly, participants who reported successful engagement with weight-loss interventions also expressed a desire to lose weight to improve health. Subthemes were identified relating to sources of motivation, and intrinsic incentives or deterrents to engagement.

#### The wake-up call

Motivation was initiated by the shock of a life-threatening cardiac event that induced a re-evaluation of lifestyle and choices (Gallagher et al., 2012b; Kramer-Kile, 2013; Nadarajah, 2012), characterized as a ***Wake-up call***.

Having a heart attack really scared me. I just wanted to feel better, see my kids grow up, and be more in control. I had tried so many things, but being in hospital really brought me to my senses. [22, p.21]

Citation 22 does not exist in the formatted references, it is Gallagher et al 2012b

Referral to CR harnessed this fear-driven motivation.

#### Self-identity

***Self-identity*** describes the range of reactions of participants to CR and individual attempts to reclaim a sense of self-identity. ACS impacted on many facets of self-identity, leaving people feeling vulnerable and acutely aware of ageing and mortality (Kramer-Kile, 2013, Nadarajah, 2012). Some faced changing roles at work or within the family due to ongoing ill-health (Kramer-Kile, 2013) or reapportioning time to accommodate CR (Nadarajah, 2012).

Engagement with CR was greatest when dietary advice aligned with the individual’s values or preferences without further compromise to an already damaged sense of self, such as accommodating extant family traditions with recommended nutritious home cooking (Gallagher et al., 2012b; Kramer-Kile, 2013). Conversely, participants who valued “alternative” lifestyle choices might try to re-assert their self-identity by resisting “mainstream” (Kramer-Kile, 2013) health advice:

Her diet fed into her larger identity as someone who was counter-cultural. … At times she became more fixated on how health professionals did not understand her rather than focusing on how her specific food choices were contributing to her weight gain. [25, p.83/84]

Citation 25 does not exist in the formatted references, it is Kramer-Kile 2013

Irrespective of whether engaging or resisting, all participants expressed commitment to prioritizing their health. No examples of unwillingness to engage in self-care or deliberate self-sabotage were identified.

#### Fulfilling social expectations

Self-identity was also a component of ***Fulfilling social expectations***. Weight loss visibly demonstrated a commitment to health improvement as they attempted to rid themselves of the social stigma of “self-inflicted” chronic disease (Kramer-Kile, 2013).

Internalized concepts of health and illness appeared to influence participants’ interpretations of CVD (Génolini et al., 2013; Kramer-Kile, 2013). Many failed to comprehend the chronic nature of CVD and described unrealistic expectations of what CR could achieve. Behaviour change was for some seen as atonement for past transgressions, to reverse damage, and regain a previous healthy state (Gallagher et al., 2012b; Kramer-Kile, 2013). The CR programmes aimed to reduce participants’ cardiac risk-factors through behaviour change (Gallagher et al., 2012a, 2012b; Génolini et al., 2013; Kramer-Kile, 2013; Nadarajah, 2012) but focus on health benefits sometimes led participants to believe they could reverse their chronic illness (Kramer-Kile, 2013; Nadarajah, 2012).

CR participants described attempting to undo previous unhealthy behaviours by engaging in diet change. The final goal was often focused on removing the disease itself. [25, p.112]

Citation 25 does not exist in the formatted references, it is Kramer-Kile 2013

Participants’ perceptions of “healthy” and “diseased” as binary states suggest discord with clinicians’ understanding of chronic illness.

#### Seeing progress

Some accepted their diagnosis and reconceptualized the meaning of health by working towards living well with chronic disease (Gallagher et al., 2012b; Kramer-Kile, 2013; Nadarajah, 2012). Others became disillusioned with both CR and their own bodies as the limitations to their recovery became apparent (Kramer-Kile, 2013). Seeing and measuring improvement through attainable goals appeared to maintain motivation (Gallagher et al., 2012b; Kramer-Kile, 2013; Nadarajah, 2012), hence the subtheme ***Seeing Progress***. Failure created despondency.

Weight-loss gave the participants a feeling of accomplishment in managing their cardiac disease. [26, p.78]

Weight-loss represented a highly visible and desirable measure of change, sustained motivation and brought social, psychological, and health benefits.

### Theme 2. Building capacity

The second theme links to the capability component of COM-B. Participants’ capacity to identify and enact healthy lifestyle changes was targeted in three ways: 1) increasing exercise tolerance; 2) improving nutritional knowledge; and 3) developing strategies to sustain changes.

#### Feeling safe

Five of the seven CR programmes described in the studies included exercise sessions supervised by clinically trained personnel (Table III). The subtheme ***Feeling safe*** emerged from many participants expressing a reluctance to exercise without supervision for fear of precipitating another event (Gallagher et al., 2012a; Kramer-Kile, 2013; Nadarajah, 2012). Exercise sessions were well received, with participants in all studies bar one (Génolini et al., 2013) preferring to increase exercise levels rather than make dietary “sacrifices”. Some found the time commitment burdensome, and some female participants expressed reservations about the male-dominated gym environment, but these tended to be outweighed by the motivational benefits of exercising among “cardiac” peers (Nadarajah, 2012).

Every one of the participants described CR as a safe place to exercise since their heart was monitored by the CR staff. The monitoring helped participants to be more self-confident. Most of the participants benefited from the peer group support at the CR setting. [26, p.86]

Citation 26 does not exist in the formatted references, it is Nadarajah 2012

The benefits of the exercise component of CR programmes were limited for those participants who struggled to develop the necessary self-confidence or motivation to continue beyond the programme (Gallagher et al., 2012b; Kramer-Kile, 2013; Nadarajah, 2012).

Improving participants’ knowledge of healthy eating was generally the most contentious intervention, often considered “wrong” by participants who had received conflicting information from other sources, or who held strong beliefs about the cardioprotective benefits of certain diets (Gallagher et al., 2012b; Kramer-Kile, 2013). To change behaviour, participants had to be open to recognizing past choices as damaging. A non-judgemental, supportive environment facilitated openness to change (Génolini et al., 2013). Personalization appeared beneficial as advice was better received when aligned with individual health beliefs and social values (Génolini et al., 2013; Kramer-Kile, 2013). Teaching about the body and causes of CVD also helped participants understand the required changes:

I had to accept what has happened and I had to make a lot more changes in my life. I was ready for it, I just didn’t know what to do because I was already eating properly … . I was on a good diet program, but I’ve improved it since. [25, p.83]

Citation 25 does not exist in the formatted references, it is Kramer-Kile 2013

Various interventions targeted the development of strategies and problem-solving skills. Some participants reported developing self-advocacy skills, linking the notion of ***Feeling safe*** as the supportive CR environment instilled confidence to speak up (Gallagher et al., 2012b; Kramer-Kile, 2013). Participants who felt overwhelmed by the complexity of problems benefitted from breaking them into manageable steps (Gallagher et al., 2012a, 2012b; Kramer-Kile, 2013), although this sometimes led to fixation on single aspects of their self-management, such as checking blood-sugar levels (Kramer-Kile, 2013).

Developing the capability to use the acquired self-management skills autonomously was not easy, with some participants becoming dependent on CR (Gallagher et al., 2012a, 2012b). Their degree of success in incorporating recommended changes into their lifestyle was dependent—like their degree of engagement—on the extent of assimilation of the CR health model into their ***Self-identity*** (Gallagher et al., 2012a, 2012b; Génolini et al., 2013).

What we’ve got to do is undo many, many years of habit—the program that we have here is that we’re sort of being re-programmed and it may take a little bit more time to do. [21, p.31]

Citation 21 does not exist in the formatted references, it is Gallagher et al 2012a

Developing autonomy required increased self-awareness of the personal, cultural, social, and environmental factors that impact on their behaviour. This included recognizing the dynamics of friendships and family relationships which supported or obstructed weight loss (Gallagher et al., 2012a, 2012b; Kramer-Kile, 2013; Nadarajah, 2012).

### Theme 3. Having support

This theme corresponds with opportunity in COM-B. As shown in ***Feeling safe,*** CR offers the opportunity to exercise, learn, and develop self-care skills with professional support. The peer group in CR programmes provides psychological support by sharing the experience of having had a heart attack and a safe space to voice the accompanying emotions. Friendly competition during gym sessions and weigh-ins boosted motivation.

...just be able to talk to other people who are facing the same issues—it makes such a big difference. [21, p.32]

Citation 21 does not exist in the formatted references, it is allagher et al 2012a

Participants whose families demonstrated proactive support reported an easier transition than others. Setting an example to help younger family members avoid future disease could be highly motivational (Gallagher et al., 2012a, 2012b; Kramer-Kile, 2013; Nadarajah, 2012). When family members were not invested in change, mealtimes proved a big challenge to participants’ compliance (Gallagher et al., 2012b; Kramer-Kile, 2013; Nadarajah, 2012). Sabotage by family members of attempts to follow guidance was surprisingly common (Gallagher et al., 2012b; Kramer-Kile, 2013; Nadarajah, 2012). This sometimes involved bonding over “forbidden” treats, but was more often linked to partners’ own ***self-identities*** as family providers of food, or cultural identities connected to the meaning of food and unwillingness to adapt culinary traditions. Illness represented an assault on these identities if diet was identified as a contributory factor. Where an individuals’ self-identity could not adapt, dietary changes were resisted.

According to Anthony’s account, his wife also shared a similar idea that changing her family’s meal preparation would also dissociate her from her identity as an ‘Italian woman.’ … Although she initially tried to support her husband’s efforts, she also had to face the implications of new knowledge that implied her commitment to an Italian diet had contributed to her husband’s health issues. [25, p.96]

Citation 25 does not exist in the formatted references, it is Kramer-Kile 2013

Complex issues around the social significance of food sometimes led to re-evaluation of relationships (Kramer-Kile, 2013, Nadarajah, 2012). Some participants reported a tension between maintaining certain relationships at the cost of their health, or risking their loss by pursuing the lifestyle changes necessary for weight-loss. Problem-solving and development of self-advocacy skills helped some participants to navigate these challenges (Kramer-Kile, 2013, Nadarajah, 2012).

Socially isolated participants had sometimes formed habit or “dependency” relationships with certain favourite foods; these provided psychological support and could prove impossible to relinquish (Kramer-Kile, 2013).

### Theme 4. Having control

This theme corresponds to the COM-B component opportunity. It interrelates with autonomy and self-confidence inherent in **Building capacity** but also includes socioeconomic issues that were outside the remit of individual CR programmes but were nevertheless influential in terms of compliance or adherence with advice.

Control over work and economic pressures were challenging for many of the participants. Attending CR incurred costs. Long working hours left little time or energy for exercise, and those in lower paid jobs could ill-afford home gym equipment or health club membership (Kramer-Kile, 2013, Nadarajah, 2012). Financial insecurity increased stress (Kramer-Kile, 2013).

Participants expressed a desire to regain control after the vulnerability and disruption caused by ACS, beginning with control over their body (Gallagher et al., 2012b; Kramer-Kile, 2013). Perceived lack of self-control was a source of frustration (Kramer-Kile, 2013) leading some participants to develop strategies to avoid or overcome temptation, such as by taking control of the shopping and cooking in their home environment (Gallagher et al., 2012b; Kramer-Kile, 2013).

## Discussion

The aim of this theoretically driven realist review was to explore which interventions successfully help cardiac patients to lose weight, which do not, and how services could be improved. All identified studies took place in a CR setting. The results extracted from the five qualitative studies reporting the experiences of 117 CR patients were synthesized and analysed using the COM-B framework (Michie et al., 2011). Further interrogation using a realist perspective data framework generated four themes that aligned with the components of the COM-B model: *Wanting to change; Building capacity; Having support; Having control*. While fear of death and/or ongoing chronic disease following an ACS event generated a desire to lose weight, both to improve health and to fulfil social expectations, the translation of this motivation into tangible weight-loss was mediated by various personal, social, and environmental influences. Self-identity, the nature of the proposed lifestyle changes and the rate of progress towards health goals affected whether attempting weight loss was deemed worthwhile. CR offered a safe place to develop physical fitness, and the knowledge and skills to make lifestyle changes. Social support and participants’ capacity to control and manage their time and environment ultimately influenced the extent to which behaviour changes could be implemented in the real world.

### Comparison with weight management studies in the general population

Two larger systematic reviews of qualitative research exploring weight-loss experiences among people without specified comorbidities (Garip and Yardley (Garip & Yardley, 2011) 17 studies, 290 participants; (Greaves et al., 2017); 26 studies, 710 participants) generated a number of findings that resonate with this review, adding confirmability (Lincoln & Guba, 1985). Yet there were some noteworthy differences in the cardiac studies included in this review.

#### Self-identity and social expectations

In both the cardiac and general population studies, self-identity and social expectations were significant for motivating participants. Social acceptance is critical to self-identity as individuals internalize and replicate social expectations and values (Shilling, 2003). In Western societies there is an expectation that individuals bear at least part of the responsibility for looking after their health to avoid becoming a burden, and slimness is associated with healthiness (Shilling, 2003, 2016). Weight-loss in those participating in CR visibly demonstrated engagement with self-care and commitment to improving personal health in accordance with social expectations. Garip and Yardley (Garip & Yardley, 2011) found self-image could shift to accommodate positive new identities, such as “runner” or “home chef”, creating an opportunity for participants to focus on how they would like to see themselves, a finding absent from the cardiac studies included in this review. Common across all studies were psychological factors (Gallagher et al., 2012b; Garip & Yardley, 2011; Nadarajah, 2012; Greaves et al., 2017; Kramer-Kile, 2013) such as developing the mental preparedness to integrate new behaviours into lifestyle, experiencing cognitive fatigue from the constant effort required, and the perpetual need to find new sources of motivation.

#### Professional support

The general population reviews found some participants were reluctant to exercise because of feeling unsafe due to neighbourhood security issues or perceived social judgement (Garip & Yardley, 2011; Greaves et al., 2017). These were not concerns for CR participants when they had access to supervised gym facilities. While many participants valued the peer and professional support available through weight loss or CR programmes, some perceived dietary guidance or exercise regimes as an imposition which reduced their personal control (Greaves et al., 2017; Kramer-Kile, 2013).

#### Strategies and sabotage

Accounts of family support and sabotage by family members were present throughout (Gallagher et al., 2012b; Garip & Yardley, 2011; Nadarajah, 2012; Greaves et al., 2017; Kramer-Kile, 2013). Building capacity by developing the mental strategies to regulate dietary intake or manage temptation was described (Gallagher et al., 2012b; Garip & Yardley, 2011; Greaves et al., 2017; Kramer-Kile, 2013). These included strategies to avoid emotional eating by finding alternative sources of comfort or adopting stress relief techniques such as relaxation or distraction (Garip & Yardley, 2011; Greaves et al., 2017).

### Qualitative synthesis as context for quantitative findings

The findings of this qualitative synthesis contribute added understanding to interpretations derived from quantitative weight management studies in cardiac populations.

#### Exploring weight change causation

Previous studies of weight loss for cardiac risk management (Abed et al., 2013; Gomadam et al., 2016; Pathak et al., 2015) noted weight gain within intervention groups but did not explore causation. Comparable results from a randomized controlled trial which evaluated community-based risk factor modification interventions (smoking cessation, physical activity, weight management) in addition to CR after hospitalization for CVD (Minneboo et al., 2017) were re-analysed in a substudy by (Tijssen et al., 2021) which identified determinants of successful weight loss among participants. The review presents insights which may support and explain some of Tijssen’s findings.

Determinants of successful weight loss included being motivated to start the programme directly after the baseline visit (Tijssen et al., 2021), recalling participants in the qualitative studies who experienced their cardiac event as a wake-up call (Gallagher et al., 2012b; Kramer-Kile, 2013; Nadarajah, 2012). The wake-up call was biographically disruptive (Bury, 1982) challenging a self-image of being healthy (Kramer-Kile, 2013). A proactive response served both to rectify a temporary loss of control over both self-image and the body.

#### Personalization and cultural sensitivity

Lower education levels were identified by Tijssen (Tijssen et al., 2021) as a determinant of weight loss among participants in the WeightWatchers© intervention. Despite qualitative findings around value placed on personalization of services (Génolini et al., 2013; Kramer-Kile, 2013), one study observed that people from lower socioeconomic groups (SEGs) more commonly attended structured weight management programmes whilst those in higher SEGs sought out self-management or personalized programmes (Génolini et al., 2013). Whether this reflects self-selection or attitudes to patients from lower SEGs held by referring clinicians (Génolini et al., 2013), differing attitudes to professional expertise (Gallagher et al., 2012a, 2012b) or attempts by patients to reclaim self-identity and autonomy through negotiation and personalization (Gallagher et al., 2012b; Kramer-Kile, 2013) are all worth exploring in future studies.

Links between food and cultural identity were described by some participants in the qualitative studies (Gallagher et al., 2012b; Kramer-Kile, 2013; Nadarajah, 2012). Sociocultural food practices communicate identity and can influence adoption or rejection of recommended dietary advice. Food prohibitions, for example, exist in most cultures and are associated with (some) religious practices. This can make them difficult to alter or relinquish even when recognized as damaging to health, as in the case of the “Italian woman” identity of Anthony’s wife (Kramer-Kile, 2013). The cultural significance of certain foods for members of immigrant communities in these studies (Gallagher et al., 2012b; Kramer-Kile, 2013; Nadarajah, 2012) was highlighted. This may provide a partial explanation for why participants who identified as being of non-Caucasian heritage disproportionately experienced weight-gain in this European study (Tijssen et al., 2021).

#### Collateral effects of risk management

Simultaneous referral to smoking cessation services was identified as a cause of weight gain in the intervention arm (Tijssen et al., 2021). The link between smoking cessation and weight gain is well documented (Bush et al., 2016), but the health benefits far outweigh any risks posed by resulting weight gain (Sahle et al., 2021). Body composition assessments as an alternative measure to weight might better communicate the contribution of exercise-induced increases in lean tissue mass to apparent weight gain. None of the qualitative studies included in this analysis discussed potential interaction between risk factor modification interventions as sources of weight gain and this would be an interesting area for future study.

### Limitations

As with all research, the findings of this review are shaped by the authors’ own priorities, values, and assumptions. As healthcare professionals, ourselves we recognize the influence of current health beliefs surrounding overweight/obesity and individual responsibility for health on our perspectives and decision-making in the review process. The choice of theoretical models used in the review has been influenced by our geographical location, as the COM-B model and realist synthesis method are prevalent in the UK but perhaps less familiar in other countries.

Thematic synthesis is a widely used method recommended by Cochrane for synthesizing qualitative evidence (Cochrane Thematic Synthesis, 2024). Realist synthesis aims to take an additional step by drawing usable conclusions. The robustness of any thematic synthesis will depend on the number of studies from which findings are drawn, and on the methodological quality of those studies (Thomas et al., 2017).

This review found a significant degree of consistency with studies of weight-management in the general population suggesting transferability, but further research is needed to verify findings specific to the target population. That said, the limited available qualitative data sourced relating to weight-management in cardiac patients and the quality of reporting (Génolini et al., 2013) may limit confirmability (Lincoln & Guba, 1985).

Qualitative designs commonly adopt purposive sampling. The accounts heard in the included studies are from CR attendees. There is evidence that those experiencing social inequalities are less likely to attend CR, including women, older people, those living in rural areas, and those without medical insurance (Resurrección et al., 2019). These voices remain underrepresented in research, perpetuating the cycle of inequality and exclusion. Anxiety and depression strongly affect CR enrolment and completion (Herber et al., 2017; Sahle et al., 2021) but were rarely discussed as a limitation in the included studies. Indeed, Nadarajah (Nadarajah, 2012) purposely excluded patients exhibiting higher anxiety levels. Symptoms of anxiety and depression affect up to 70% of ACS patients and are associated with worse cardiac prognoses (Feng et al., 2016; Lane et al., 2003; Serpytis et al., 2018). Insight into the role of mental wellbeing on motivation and self-efficacy in cardiac patients could have significant implications for weight management.

The use of the COM-B model, itself a synthesis of behaviour change theories (Michie et al., 2011), arguably limits the ability to explain variability in behaviour through over-systemization, thereby leaving gaps in the narrative (Ogden, 2016). Used as a framework to seek out these gaps as avoidable “pitfalls” [Peters & Kok, 2016; p. 265], COM-B can help illuminate areas where services are not achieving behaviour change. The COM-B model has been criticized for over-emphasizing the role of individual behaviours in health outcomes whilst overlooking socioeconomic and structural determinants of health (Ogden, 2016). This argument may be countered by positioning COM-B within its correct theoretical context as part of the behaviour change wheel. The outer rings of the behaviour change wheel offer higher-level options to influence behaviour, such as social policy and fiscal measures (Michie et al., 2011). While these options remain inaccessible to most health care professionals and of little practical help in altering harmful health behaviours at a patient-facing level, COM-B is increasingly being adopted to guide health and social policy in the UK. Using an extensively validated framework familiar to policymakers as a foundation for this review contributes to its usability.

### Implications for practice

Thematic synthesis aims to identify clinically usable findings. The recommendations made on the basis of this review are limited by the scarcity of available data as discussed above. Further research is needed to guide service development and management especially where there are cost implications. Our findings may be useful for guiding individual clinical practice and considering the content, structure, timing, and accessibility of CR and weight management support for patients with heart disease.

The finding that patients reported high motivation to make healthy lifestyle changes in the immediate post-MI period supports inpatient referral to CR programmes as per current practice. Adequate staffing and resources are vital to optimize service capacity. Minimizing the financial impact of CR for attendees by offering flexible hours to fit work schedules and mitigating transport costs could facilitate participation. Alternative approaches to motivate patients should be explored, especially for those whose heart disease does not entitle them to CR under current service provision, or who have not experienced an acute “wake up call” event.

Participants in CR viewed weight loss as a positive indicator of their progress. Whilst offering encouragement, practitioners should be aware of lay health beliefs and avoid reinforcing notions that lifestyle changes can reverse or remove disease. Focus on risk management and living well with heart disease may help manage expectations and avoid despondency leading to renunciation of healthy lifestyle changes should weight loss prove elusive.

Given the significance of self-identity and bodily limitations throughout the findings, the development of personalized programmes which consider participants’ needs, views, and preferences could make weight management attractive and more effective for a broader population. The findings of this review suggest programmes should include individualized exercise programmes, consistent advice presented as easy-to-follow steps, and assistance to develop problem-solving skills, strategic thinking, and psychological preparation to exercise outside the “safe” CR environment. Ongoing access to suitable and acceptable exercise facilities might be enabled through the development of commercial partnerships.

By fostering an awareness of the social context within which patients operate, health care workers can gain insight into the potential limitations of CR programmes and assist individuals to focus on the most sustainable and achievable lifestyle changes for them. Training in techniques such as Motivational Interviewing (Miller & Rollnick, 2023) may assist this process (Vellone et al., 2020).

### Further research

Risk assessment and clinical management of people with CVD has long included weight-management (Hippisley-Cox et al., 2008) yet little research evaluates the success of interventions or patients’ experiences. Further research into patients’ needs, preferences and perceptions, and potential interactions between risk modification interventions could identify underlying mechanisms to inform intervention design and improve care.

## Conclusions

Most cardiac patients with overweight/obesity express a desire to lose weight when participating in CR. Services aimed at facilitating healthy lifestyle improvements are offered to patients with CVD, but the requisite changes may prove difficult to achieve for an array of interconnecting psychological, social, cultural, and economic reasons. Weight management and CR programmes that accommodate the patient’s disrupted self-identity and adapt to meet the needs of individuals are most acceptable, but have little influence over external social and socioeconomic factors affecting weight management. Providing a wider choice of support and interventions to CR patients may increase the proportion who successfully manage their weight. Research is needed to understand interactions between weight management and concurrent risk factor modification interventions such as smoking cessation, and to explore the weight management experiences of patients with other cardiac pathologies.

## Supplementary Material

Appendix1_CardiacWeightManagementTS2024.docx
