# Supplementary material for: Barriers and facilitators to weight-loss in patients with overweight/obesity and cardiac disease: a realist qualitative synthesis
Source: Int J Qual Stud Health Well-being. 2024 Oct 26;19(1):2419574. doi: 10.1080/17482631.2024.2419574 (PMC11514393; doi:10.1080/17482631.2024.2419574)
Supplement: Appendix1_CardiacWeightManagementTS2024.docx [file ZQHW_A_2419574_SM0100.docx]

**Appendix 1.**

**Literature Review Protocol**

1. **Background**

This review aims to explore the literature addressing the views and experiences of people with heart disease and obesity regarding healthy weight management.

The link between atrial fibrillation and obesity is becoming increasingly well established in the literature, but a scoping review on Google Scholar found no research into the implications of this link for the care of people with a diagnosis of AF, nor of attempts to disseminate this knowledge among patients. Searches of atrial fibrillation websites aimed at professionals and patients similarly found no information about the management of obesity and atrial fibrillation (Arrhythmia Alliance, Atrial Fibrillation Association, Atrial Fibrillation Network, searched on 24-Sep-2019).

The link between obesity and risk factors for AF such as hypertension, coronary artery disease, Type 2 Diabetes Mellitus and Obstructive Sleep Apnoea is similarly well documented in the literature (Abed *et al*, 2013; Pathak *et al*, 2015; Lavie *et al*, 2017). Literature reviews have been carried out exploring the views and experiences of people with obesity to weight management (Garip and Yardley, 2011). No reviews relating to AF and obesity were identified by the scoping review.

1. **Objective**

To identify and evaluate the literature exploring patient perspectives and experiences of weight management with a heart condition. Particular attention will be paid to connections between the diagnosis and the heart condition, and to what sort of help and advice has been received or would be valued from health care professionals.

1. **Review Question**

**3.1** The question to be addressed will be: What are the views and experiences of people with obesity and heart disease regarding weight management?

**3.2** The PICo (Population, Interest, Context) tool will be used to elaborate the search terms from this question (see table 1).

**Table 1. PICo**

| Population | Interest | Context |
| --- | --- | --- |
| People with heart disease and obesity  Search terms:  Heart  Heart disease  Heart Condition  Cardiac  Cardiovascular  Overweight  Obese/Obesity/Obes*  Arrhythmia  High BMI | Views and experiences  Search terms:  Views  Opinions  Attitudes  Perspectives  Perceptions  Barriers  Obstacles  Challenges  Facilitators  Experiences  Want/desire  Difficulty | Weight management  Search terms:  Weight management  Weight loss  Dieting  Slimming  Exercise  Lose/losing weight  Weight control  Healthy weight  Bariatric surgery  Gastric band/balloon/bypass |

1. **Evidence Gathering and Selection**

**4.1** **Evidence gathering**

The evidence gathering approach will have four components:

**4.1.1** The following databases will be searched:

- CINAHL
- Medline
- PubMed
- PsycInfo

**4.1.2** Websites of organisations specialising in AF will be hand searched, including:

- AFNET <https://www.kompetenznetz-vorhofflimmern.de/en/>
- Heart Rhythm Alliance <http://www.heartrhythmalliance.org/>
- British Heart Foundation <https://www.bhf.org.uk/>

**4.1.3** Grey literature will be searched via

- Grey Matters search tool <https://www.cadth.ca/resources/finding-evidence/grey-matters>
- HMIC Health Management Information Consortium
- Conference proceedings from large arrhythmia conferences, including
  - European Society of Cardiologists Congress
  - Heart Rhythm Congress
  - World Heart Rhythm Conference

**4.1.4** The references of papers which meet the eligibility criteria will be hand-searched

**4.2** Eligibility

**4.2.1** Types of studies. Studies examining patients’ experiences and opinions will be included. These are expected to be predominantly qualitative studies involving structured or semi-structured interviews, focus groups, patient diaries etc. Studies based on questionnaires may also be included.

**4.2.2** Types of participant. People who are overweight or obese as defined by the study, or who have been in the past but have successfully lost weight, who have been diagnosed with any cardiac condition. If an insufficient number of papers is identified, these criteria will be expanded to include recognised cardiac risk factors and comorbidities, including Type 2 Diabetes Mellitus, hypertension, obstructive sleep apnoea.

**4.2.3** Types of intervention. No interventions will be specified. All professional and personal weight management strategies, advice, weight management programmes, diet and exercise regimens, bariatric surgery etc will be included.

**4.2.4** Outcome measures will include but not be limited to opinions, views, experiences, perspectives, perceptions, successes and failures, attitudes, wants, desires, needs, satisfaction scores.

**4.3** Exclusion criteria

- Studies examining the opinions and experiences of health care professionals who deal with people with obesity and heart conditions.
- Editorials, opinion pieces, letters etc written by health care professionals
- Studies measuring the efficacy of weight management interventions
- Studies where the full text is not available for evaluation will be excluded

The number of excluded papers and reason for exclusion will be recorded at each stage.

A PRISMA diagram will be used to illustrate the evidence gathering process.

**5. Evaluation of the evidence**

Studies will initially be evaluated by title and abstract to assess relevance. Where this does not provide enough evidence a full-text evaluation will be carried out.

The full text of all studies meeting the eligibility criteria for inclusion in the review will be read and evaluated using the CASP tool.

A summary of the results of the evaluation will be presented in table form.

The results will be synthesised into a literature review to underpin a qualitative study of the views and experiences of people with obesity and AF relating to healthy weight management.

**6. Management and referencing of sources**

A spreadsheet will be kept recording search terms and numbers of papers identified from each source.

Eligible papers will be saved and managed using the Mendeley Reference Management Software.

**References**

Abed HS, Wittert GA, Leong DP, Shirazi MG, Bahrami B, Middeldorp ME, Lorimer MF, Lau DH, Antic NA, Brooks AG, Abhayaratna WP, Kalman JM, Sanders P. Effect of weight reduction and cardiometabolic risk factor management on symptom burden and severity in patients with atrial fibrillation: a randomized clinical trial. JAMA. 2013 Nov 20;310(19):2050-60. <https://doi.org/10.1001/jama.2013.28052> PMID: 24240932.

Pathak RK, Middeldorp ME, Meredith M, Mehta AB, Mahajan R, Wong CX, Twomey D, Elliott AD, Kalman JM, Abhayaratna WP, Lau DH, Sanders P. Long-Term Effect of Goal-Directed Weight Management in an Atrial Fibrillation Cohort: A Long-Term Follow-Up Study (LEGACY). J Am Coll Cardiol. 2015 May 26;65(20):2159-69. <https://doi.org/10.1016/j.jacc.2015.03.002> Epub 2015 Mar 16. PMID: 25792361.

Lavie CJ, Pandey A, Lau DH, Alpert MA, Sanders P. Obesity and Atrial Fibrillation Prevalence, Pathogenesis, and Prognosis: Effects of Weight Loss and Exercise. Journal of the American College of Cardiology 2017 70;16:2022-2035 <https://doi.org/10.1016/j.jacc.2017.09.002>.

Garip G, Yardley L. A synthesis of qualitative research on overweight and obese people's views and experiences of weight management. Clin Obes. 2011 Apr;1(2-3):110-26. <https://doi.org/10.1111/j.1758-8111.2011.00021.x>. PMID: 25585576.
